# Nanoparticles: Oral Delivery for Protein and Peptide Drugs

**DOI:** 10.1208/s12249-019-1325-z

**Published:** 2019-05-20

**Authors:** Shu-jun Cao, Shuo Xu, Hui-ming Wang, Yong Ling, Jiahua Dong, Rui-dong Xia, Xiang-hong Sun

**Affiliations:** 1 Pharmacy College of Qingdao University, Qingdao, 266021 China; 2 Stomatology College of Qingdao University, Qingdao, 266021 China; 3 Affiliated Hospital of Qingdao University, Qingdao, 266555 China

**Keywords:** protein and peptide drugs, nanoparticles, oral delivery, bioavailability

## Abstract

Protein and peptide drugs have many advantages, such as high bioactivity and specificity, strong solubility, and low toxicity. Therefore, the strategies for improving the bioavailability of protein peptides are reviewed, including chemical modification of nanocarriers, absorption enhancers, and mucous adhesion systems. The status, advantages, and disadvantages of various strategies are systematically analyzed. The systematic and personalized design of various factors affecting the release and absorption of drugs based on nanoparticles is pointed out. It is expected to design a protein peptide oral delivery system that can be applied in the clinic.

## INTRODUCTION

Since the first bioactive peptide was synthesized in 1953 by Robert Bruce Merrifield, the research of protein and peptide drugs has been developing rapidly, and the research of protein and peptide drugs targeting multiple receptors has been carried out in the world. Protein and peptide drugs have many valuable applications in the clinic to treat or prevent diseases by modulating physiological or pathological processes. And protein and peptide drugs play an indispensable role in cancer, autoimmune diseases, and cardiovascular diseases; especially in the field of tumor and diabetes treatment, many protein and peptide drugs have been listed, and great economic benefits have been achieved (1). However, their widespread application is restricted due to chemical and physical instabilities, such as low pH value, enzymatic degradation in the gastrointestinal tract, and rapid elimination from circulation in contrast to small-molecule drugs (2).

The destruction of gastric acid, the degradation of digestive enzymes, and the mechanical barrier that is difficult to cross the biofilm affected the absorption of drugs. Proteins and protein and peptide drugs can only be administered in the form of intravenous and subcutaneous injection, and often need long-term repeated administration, which brings great inconvenience to patients (3). Therefore, the development of oral protein drugs is of great significance. However, high-risk infection and poor patient’s acceptability and compliance for chronic disease have limited their clinical use. Oral delivery has advantages over other forms of delivery which lead to better patient acceptability and reduce frequency and painless administration, which can help in better disease management (4). However, oral delivery is restricted due to its low bioavailability. The main reasons are drug degradation by stomach acids and proteases in the digestive system and a drug’s inability to cross intestinal membrane barriers. Even if the drug arrives at the gastrointestinal tract (GI), it still faces obstacles as regards stability and absorption (4). Therefore, the most important problem for orally administered protein and peptide drugs is improving bioavailability.

The development of new drug formulations and new technologies has become a way to improve the utilization of protein and peptides drugs; nanotechnology has promoted the clinical application of protein drugs in recent years (5). And the advantages of nanoparticles as protein and peptide drugs carriers are listed in Table I (1). However, nanoparticles are rarely given with proteins and peptide drugs, and these drugs are still in the early stage of research and development.

**Table I:** Advantages of Nanoparticles as Protein and Peptide Drug Carriers (1)

1. Reduces the enzymolysis and aggregation of protein and peptide drugs in the gastrointestinal environment and increases the transmembrane absorption of the small intestinal epithelium.	
2. Changes the distribution of the drug in the body	
3. Both preparation material and preparation process are simple	
4. Achieves the therapeutic effect of controlled release and target to treat diseases	
5. Be targeted by the modified target ligand and prolong the retention time at a specific absorption site.	

## THE ABSORPTION MECHANISM OF NANOPARTICLES AS CARRIERS FOR PROTEIN PEPTIDE DRUGS

Generally speaking, protein and peptide drug–loading nanoparticles have four ways through the gastrointestinal membrane, which are transmembrane transport, receptor-mediated transport, vector-mediated transport and M cell(membranous/microfold cell) transport (6).

The transmembrane transport pathway is that nanoparticles enter the cell by using the endocytic mode and then pass through the cell basement membrane and are released into the body circulation. Intestinal epithelial cells and M cells are the most important transmembrane transport cells in the gastrointestinal tract (7). M cells are microfold cells located in the intestinal collecting lymph nodes, which contain M cell capsules and Peyer’s lymphoid aggregation (8). The surface of M cells can absorb particles and transport antigens from intestinal cavity to lymphoid tissue, thus inducing a mucosal immune response (9). It is generally believed that the uptake of the nano-drug delivery system mainly occurs in M cells or cytosolic. The efficiency in the uptake and transport of the nano-drug delivery system by M cells are significantly higher than that of intestinal cells. However, glycosyl arrangement lacks a complete glycocalyx structure, and the apical membrane microvilli are sparse, which is conducive to the contact and fusion of drug particles with cells (10). Protein macromolecules can selectively adhere to the corresponding glycoproteins and thus be absorbed by M cells. At the same time, there are depressions in the cell membrane on the lateral basal surface, which make it a natural place for lymphocytes, dendritic cells, and phagocytes to gather and stay. It can effectively shorten the distance of drug particles being transported across the membrane into the systemic circulation. Therefore, the transmembrane transport function of M cells is a potential pathway for oral nanoparticle protein and peptide to pass through the gastrointestinal tract.

Receptor-mediated and carrier-mediated transports bind to the corresponding ligands through receptors on the membrane or intramembrane carriers, respectively, and then complete by phagocytosis or cytokines, which have the advantages of high efficiency and selectivity. Receptor-mediated endocytosis is not restricted by the size of the drug molecule but by the type of receptor. The ligands identified include lectins, toxins, vitamins, and transferrin (5). Carrier-mediated transports mainly target small molecules or oligopeptides, such as monosaccharides, angiotensin-converting enzymes, and amino acids*.* (11) According to the characteristics of these two transport pathways, the permeability of drugs can be increased by modifying ligands in the structure of protein polypeptide drugs and by binding with receptors on intestinal cell membranes (12).

Nanoparticles have the following absorption mechanism in the gastrointestinal tract: (13–15) the nanoparticles can translocate the intestinal lymph node (Peyer’s patches) *via* endocytosis, and then the endocytosis and translocation of M cells in the intestinal lymph nodes. The nanoparticles in the intestinal cavity are mainly distributed in the apical region of M cells and then engulfed by M cells by endocytosis. Compared with normal epithelial cells, M cells reduce the activity of membrane hydrolase and affect the uptake of protein or protein-modified nanoparticle (16). Damge *et al* (17) prepared the interfacial polymerization of insulin cyanoacrylate nanoparticles after oral administration, a hypoglycemic effect can be maintained for 20 days. Compared with the intravenous injection, the bioavailability of the dissolved drugs was 22%, while the oral bioavailability of the nanoparticles was 36%. Wang M *et al* reported that the absorption of optimized chitosan nanoparticles was enhanced by 4.7-fold in MDCK cell monolayers and by 2.0–2.78-fold in different rat intestinal segments, with no significant difference between the duodenum, jejunum, and ileum compared with free exendin-4 (18).

Although the oral route is a very hostile environment for peptide and protein drugs, most of the nanoparticle delivery system can help overcome many of the barriers encountered in following oral administration including enzymatic degradation and poor membrane permeability (11,19). There are some specific tissues related to immunity in the intestines of humans and animals, such as Peyer’s patches. This area can account for about 25% of the whole intestinal mucosa, and it is the main site of the uptake of nanoparticles (20). Nanoparticles are taken or transferred into the blood circulation, which can also enhance uptake by M cells in Peyer’s patches, and this may be the main way for nanoparticles to be taken. Because of its small size, increased surface area, and high adhesion to the biofilm, nanoparticles will accumulate in the Peyer’s junction when they enter the intestine (20–22). .They will carry biological macromolecules through the biological mucosa in a complete form, thereby improving the bioavailability of oral drugs.

Based on the above gastrointestinal transport mechanism and absorption characteristics, it is a promising research strategy to prepare protein peptides into an oral nanoparticle delivery system to improve its bioavailability. In general, micron-level particles can be absorbed by Pyle’s lymph nodes. Therefore, oral nanoparticles can be targeted to the gastrointestinal tract of the Pyle collection of lymph nodes, through the transmembrane transport and absorption into the systemic circulation, and the use of a special carrier material can be prepared, such as bioadhesive enteric release functional microspheres, effectively improve the bioavailability of the protein polypeptide drug.

## BARRIERS TO ORAL DELIVERY OF PROTEIN AND PEPTIDE DRUG NPs

**Fig. 1:**
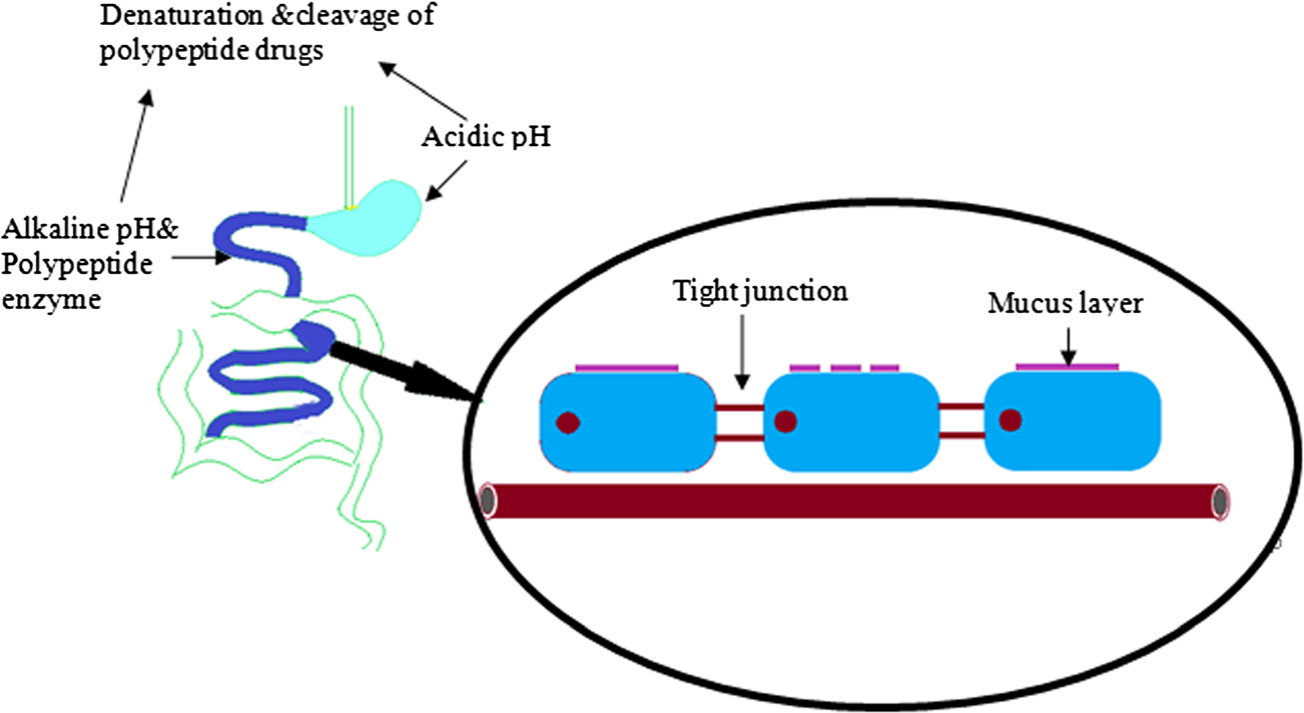
Major barriers to oral delivery of peptide and protein-based drugs

Most of the protein and peptide drugs are macromolecular hydrophilic, in the extreme environment of gastrointestinal stability, susceptible to gastric acid and pepsin degradation; at the same time, because of its hydrophilicity, it cannot effectively penetrate the physiological barrier of the small intestinal mucous layer and epithelial layers such as the intercellular tight junction (Fig. 1). Most of the strategies for oral administration of protein and peptide drugs are the same, that is, to avoid enzymatic degradation in the digestive system, and to improve the drug oral bioavailability (23).

### Mucus Barrier

Mucus is composed of mucin, enzymes, electrolytes, and water, which acts as a lubricant and protects the intestine (24,25). Mucus can quickly capture and eliminate external particles through adhesion and space barriers to prevent pathogens and various toxins from invading mucous epithelial cells, and it also hinders and restricts the effective diffusion and absorption of the drug, which makes the bioavailability of the mucous administration lower (26,27). Mucin is the main component of mucus to produce viscoelasticity, secreted by the goblet cells of the epithelial membrane propria and the serous mucin gland and consist of the main protein chain and the oligosaccharide side chain (6). Due to the electrostatic interaction and hydrogen bonding, such as salt bridge physical crosslinking, the reticular structure formed between the oligosaccharide side chains and molecular, and a part of non-crosslinked protein molecules as a soluble component between the reticular structure form a viscous state (28). The main mechanism of mucus-adhering particles is the multivalent adhesive interaction force generated by the carboxyl group and the negative charge of sulfuric acid of the oligosaccharides, followed by the high-density hydrophobic region and mucinous protein fibers on the mucin chain (6,26). And, they offer a certain level of resistance to the protein drug diffusion by the viscosity and the interactive nature of these layers (6)..

The mucus in the region of the Peyer’s patches is relatively sparse and helps nanoparticles break through the mucosal barrier and other intestinal lymphoid tissues enter the blood circulation, which is the main absorption route of oral nanoparticles (24). Hydrophobic nanoparticle is the main factor that affect the intestinal absorption of some collection of lymph nodes. Hydrophobic polymers such as polystyrene, polymethyl methacrylate and poly hydroxybutyrate, and glycolic acid polymer nanoparticles can effectively be intestinal Peyer absorption than low hydrophobic lactic acid. The absorption intensity of hydrophobic particles is about 100 times as the hydrophilicity of polymer cellulose. But hydrophobic interaction plays a major role in mucous adhesion, and it is also a major obstacle to mucous infiltration (29).

However, routine after oral administration, most of the nanoparticles do not adhere or through the mucous layer of the small intestine, the residence time in the small intestine of short, and most of the drugs do not have enough time to release directly excreted. At the same time, due to the hydrophilic layer of the intestinal epithelial tight junction protein and polypeptide, the release of the drug to be absorbed in the gastrointestinal tract, cause the nanoparticles in the low oral bioavailability.

### Intestinal Barrier

The structure of different M cells in normal intestinal cells, the surface glycoprotein containing glycosyl, but lack of the complete structure of the glycocalyx arrangement, and apical membrane microvilli became sparse, is conducive to drug particles and cell contact fusion protein molecule with selective adhesion glycoprotein accordingly, from which M cell uptake (10,30). At the same time, there is a depressed structure on the basal cell membrane, which is a natural place for lymphocytes, dendritic cells, and phagocytic cells to gather and stay. It can effectively shorten the distance between the transmembrane transport of drug particles and the circulation of the body.

Intestinal epithelial cells and M cells are the most important transmembrane transport cells in the gastrointestinal tract. The drug can be absorbed through the cross-cell channel, and it needs to be crossed by the passive diffusion, carrier assisted diffusion and vesicle transport. The cell membrane phospholipid bilayer structure which has semi-permeable, fat-soluble molecules can pass through the passive diffusion across the cell membrane, protein and polyp drugs need to be transported through the membrane through the active transport to enter the cell (31).

The tight junction of intestinal epithelial cells is the main connection between intestinal epithelial cells (28). It plays an important role in maintaining the polarity of epithelial cells and regulating the permeability of the intestinal barrier. Tight junctions form a barrier, which allows the absorption of water and electrolytes from the intestinal cavity, prevents inflammation and infection factors from entering the systemic circulation, and has important significance for maintaining homeostasis. However, tight junctions are the major barrier for large molecular weight drug permeation between the cells, and studies have shown that the lack of tightly connected cell layers is basically a barrier-free function (1,32). In the absence of any absorbent, only small molecular weight drugs can pass through.

### Enzyme Barrier

Due to the action of dissimilarity in the brush border and intracellular cytoplasm, various substrate-specific enzymes in the digestive cavity form a huge enzyme barrier. Besides, the acid and alkali environment of the digestive tract also has a great influence on the absorption of peptide drugs. Most peptides and proteins are not acid fast, while the pH value of gastric acid is 1~3. When protein and peptide drugs pass through the stomach, a part of the drugs are hydrolyzed and lose their biological activity due to the complex pH environment of the whole gut (1,33). The drug enters the system, the metabolism caused by various enzymes, especially the action of various forms of proteolytic enzymes, leads to the degradation of drugs into small peptides or amino acids. Therefore, the enzyme barrier in the digestive tract has also become a major obstacle to the absorption of protein and peptides drugs (30).

## THE INFLUENCE OF PHYSICOCHEMICAL PROPERTIES OF PROTEIN/PEPTIDE DRUG-LOADED NPs

### Nanoparticle Material

Nanoparticles, which are divided into polymer nanoparticles and solid lipid nanoparticles, have been widely applied to oral delivery of protein and peptide drugs using both synthetic and natural materials, including gelatin, hyaluronic acid, cellulose, chitosan, poly(lactic-co-glycolic) acid (PLGA), polycaprolactones, polyanhydrides, and cyclodextrins (34,35). The degradation of natural polymer materials and the rate of drug release are faster; the release rate of synthetic polymer materials is relatively slow and can last for a few days to several weeks (16). Polymeric nanoparticles can be used as the standard for judging the carrier of protein and peptide drugs (36–38): (1) it can encapsulate drugs and protect them from enzymes in the digestive system; (2) the size, shape, and distribution of nanoparticles should be consistent with the requirements; (3) the drug loading rate and entrapment efficiency were higher; (4) the release time of the drug should be well enough for the clinical medication standard; (5) the carrier material must be nontoxic and biodegradable.

### Diameter

Diameter is an important factor affecting the absorption of the nanoparticles, and only the particle size is suitable. Knowledge of NP size influence uptake by M cells and enterocytes, and NPs can preferentially translocate by endocytosis through enterocytes in sizes smaller than 50 nm, while M cells transport particles preferentially in the small (20–100 nm) but also in the larger (100–500 nm) size range (15,39). Meanwhile, there are two routes of absorption of drugs in the small intestine including the paracellular pathway and transcellular pathway.

Desai *et al* (40) prepared a series of polystyrene nanoparticles of different sizes using BSA as a model drug. The objective was to study the intestinal tract of mice with different-size nanoparticles and to study the intestinal absorption of nanoparticles with different sizes, and they drew the following conclusions: the absorption rate of 100 nm nanoparticles was 15–250 times larger than slightly larger particles; the former can penetrate the intestinal epithelial cells, while the latter is mainly distributed in the epithelial layer of the small intestine, and cannot be absorbed; particles larger than 500 nm cannot be absorbed by vesicles; in contrast, particle sizes less than 500 nm can reach the circulatory system. Hussain *et al*’s (21) study has shown that the absorption of nanoparticles in the gastrointestinal tract has an optimal particle size. In addition, the size of the diameter is too small and the preparation cost is high at the expense of drug loading. Therefore, only nanoparticles with suitable particle sizes can be prepared to meet the clinical needs.

### Surface Charge

The electrostatic interaction of positively charged nanoparticles (such as chitosan) and negatively charged mucin glycosides can lead to strong mucosal adhesion, promote nanoparticles to close to epithelial cells, and increase the intake of intestinal aggregated Peyer’s patches (41). Because of the negative charge in the mucus layer of the small intestine, the nanoparticles with a positive charge, such as chitosan nanoparticles, are more likely to adhere to the mucus layer of the small intestine and prolong the residence time of the nanoparticles in the small intestine. However, the ionic interaction between cationic nanoparticles and mucus layer hinders the further penetration of nanoparticles through the mucous layer to the surface of epithelial cells, which affects the uptake of nanoparticles by epithelial cells.

Nanoparticles with a positive surface charge are more easily removed *in vivo* than those with a negative or neutral surface charge, while the neutral surface is most suitable for prolonging the cycle time of nanoparticles *in vivo* (42). The modification of PEG on the surface of nanoparticles can not only improve the hydrophilicity of particles but also shield the surface charge of nanoparticles, which makes the surface charge of particles close to the electric neutrality. The relative molecular mass and coverage density of PEG have great influence on the surface potential of particles. Therefore, measuring the action potential of particles can indirectly characterize the degree and shielding effectiveness of PEG on the surface of nanoparticles, and predict its diffusion behavior in mucus. Wang *et al* (43) found that if the absolute value of the action potential of nanoparticles is greater than − 10 mV, it shows obvious mucosal adhesion, and proposes nanoparticles. The action potential of the particles from mucous adhesion to sticky inertia should be between – 10 and − 7 mV (pH neutral condition).

The surface charge of the carrier material affects the adhesion of the nanoparticles to the intestinal mucosa. There is a negatively charged sugar group in mucus. Therefore, carrier materials loaded with a positive charge can form electrostatic forces with mucus and prolong the retention time of nanoparticles on mucosal surfaces. However, the ionic interaction between cationic nanoparticles and mucus layer hinders the further penetration of nanoparticles through the mucous layer to the surface of epithelial cells, which affects the uptake of nanoparticles by epithelial cells.

### Surface Modification

It is understood that the surface of nanoparticles can contribute to the absorption in the intestinal mucosa, but there is no unified conclusion. However, the researchers generally agree on a point that the surface characteristics of nanoparticles play a crucial role in crossing the mucosal layer. There are many ways to increase the ability of nanoparticles to penetrate the mucosa, such as changing the physical and chemical properties of nanoparticles and adding a group on the surface of nanoparticles to increase the targeting of nanoparticles (44). Chemical modification can change the physical and chemical properties of nanoparticles, the hydrophobicity and surface charge of nanoparticles, the stability of nanoparticles, the entrapment efficiency of proteins, and the adsorption capacity of the mucosa (44). Li Wei *et al* (45) pointed out that the synthesis of two amphiphilic block copolymer PBMA-b-PAM and PBMA-b-PAM micelles stability formed in the serum is significantly different by changing the hydrophilic and hydrophobic chain length and the ratio between them. The results show that the increase of chain density in the hydrophilic shell can significantly reduce the interaction between the hydrophilic chain and serum protein, which makes the nanomaterial more stable and prolongs the cycle time *in vivo*.

PEG modification increased the stability of protein and peptide drugs, increased the solubility, reduced the immunogenicity, prolonged the residence time in blood, reduced the protease degradation, and reduced the frequency of drug use (46). And in the intestinal tract, PEG nanoparticles are mainly taken up by the Peyer’s patches on the intestinal wall, and some studies have shown that the affinity for pies is higher than that of the common nanoparticles. This phenomenon may be due to the “brush action” of the PEG chain, which makes it easier for the PEG nanoparticles to penetrate the mucilage layer (47). Yoncheva *et al* (47) reported that thiol modified acrylic polyester encapsulated insulin nanoparticles were prepared by cysteine modified acrylic polyesters. And *in vitro* mouse small intestine simulation experiments showed that the amount of adhesion of cysteine-modified nanoparticles was three times that of unmodified nanoparticles; that is because there are many free thiol groups on the surface of nanoparticles, which can interact with the glycoproteins in the mucous membrane, so that the cysteine modified nanoparticles have more mucosal adhesion than the unmodified nanoparticles. *In vivo* experiments showed that the modified nanoparticles had better adhesion to the intestinal mucosa, thereby increasing the release rate of the nanoparticles, and thus had a better hypoglycemic effect.

In addition, a lectin that can initiate endocytosis and improve drug bioavailability is prepared by covalently binding the lectin with the intestinal mucosa to the surface of nanoparticles. This is because the cells are bound to sugar groups, different cells express different glycosyl sequences, and some cancer cells secrete abnormal glycoproteins with specific sugar so that it becomes a lectin specific binding site. Using this principle, the absorption rate of nanoparticles can be improved by grafting lectin on the surface of nanoparticles.

## METHODS TO PROMOTE THE ORAL DELIVERY OF PROTEIN AND PEPTIDE DRUG NPs

**Fig. 2:**
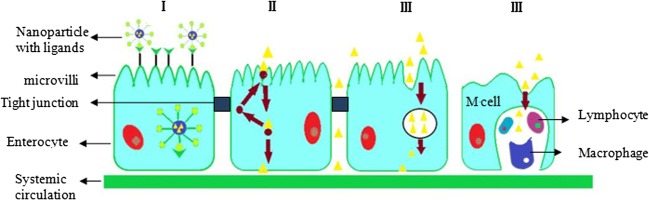
Schematic representation of the transport mechanisms: (I) receptor-mediated transport; (II) carrier-mediated transport; (III) paracellular transport; (IV) phagocytosis by M cells

Although it has been proved that the nanoparticles can be absorbed by the GT after oral administration, low absorption is the biggest obstacle to the development of oral nanoscale drug delivery systems. With the further research of absorption mechanism, many methods to promote the absorption of nanoparticle gastrointestinal tract are mainly focused on the modification of intestinal epithelial cells with adhesion and targeting on M cells. And there are four distinct mechanisms for molecules to cross the cell membrane including paracellular, transcellular, carrier-mediated, and receptor-mediated transport (Fig. 2). The strategies reported for improving the bioavailability of protein include a chemical modification, absorption enhancers, mucous adhesion systems, and nanoparticle list in Table II. This section reviews the strategies for improving the oral bioavailability of protein and polypeptide drugs.

**Table II:** Advantages and Disadvantages of Approaches for Enhancing Oral Bioavailability of Proteins and Peptides

Method	Advantages	Disadvantages
Absorption enhancers	Enhancing oral bioavailability	No protein specific and risk of toxin or allergen import along with the proteins
Enzyme inhibitors	Preventing the enzymatic degradation of protein and peptide drugs	High toxicity
Mucoadhesive systems	Prolonging retention time and improving oral bioavailability	No avoiding rapid mucus clearance and penetrating the mucus layer
Colon-specific drug delivery	Protecting the activity of protein and peptide drugs	Having technical difficulties

### Absorption Enhancers

Absorption enhancers can play a role through the cell and (or) cell bypass pathway. The mechanism of absorption enhancers is listed in Table III. The cell pathway may be an absorption enhancer, which promotes drug absorption by interfering with the outer membrane structure of the cell or causing the loss of membrane protein. The intercellular way promotes drug transport by opening tight junctions between cells. The close connection between the membrane of the epithelial cells of the gastrointestinal tract and the cells greatly restricts its oral absorption (32,36). The hydrolysis of gastrointestinal digestive enzymes and the low permeability of intestinal epithelial cells are the two major obstacles to the oral absorption of peptides and protein drugs (1,32). Therefore, the application of absorption enhancers on the nano-drug delivery system can improve peptide absorption through the intestinal membrane and the bioavailability of proteins and protein and peptide drugs to a certain extent (5,48). Permeation enhancers increase the penetration of cellular peptides, and a good tight junction regulation seems to be a more attractive way (28). The molecular weight of peptides and protein drugs is large and has strong polymerization ability. Therefore, the application of absorption enhancers can nonspecifically temporarily break the intestinal barrier by changing the cell integrity of tight junctions and enlarging intercellular spaces, or by disrupting the lipid bilayer stability to the formation of holes, so as to improve the biological membrane permeability, enhancing protein peptide drug absorption *via* the gastrointestinal tract into the blood (11,49,50).

**Table III:** The Mechanism of Absorption Enhancers

1. Temporary destruction of the integrity of the intestinal barrier	
2. Reducing the viscosity of the mucous layer	
3. Opening the close connections between the epithelial cells	
4. Increasing the fluidity of the membrane	

Makhlof *et al* (51) studied the safety penetration enhancer of the adherent particle system for the oral administration of protein and peptide drugs. Based on the ionic interaction between spermine (SPM) and polyacrylic acid (PAA), polyelectrolyte nanoparticles (NPs) were prepared. In *in vivo* oral test of rats, FD4 had strong and continuous osmosis. The blood calcium of rats decreased significantly, and NPs could effectively improve the oral absorption of calcitonin. The study of Caco-2 monolayer cytotoxicity showed that fluorescein isothiocyanate (FD4) had high permeability to SPM-PAA particles. And Lanke *et al* (48) reported that LMWH and an absorption enhancer papain were encapsulated in bovine serum albumin matrix and four formulations were spray-dried, which showed that *in vivo*, the presence of papain can significantly improve the level of drug absorption and bioavailability can reach 21%. Therefore, the balance of bioavailability and safety needs to be further improved.

However, the use of absorption enhancers can alter the risk of the intestinal biotic environment and make more systemic exposure to dietary antigens, leading to an increased risk of autoimmune diseases (52,53). The changes in the membrane of the absorption enhancers may lead to a series of problems, such as some other toxic substances that have not been absorbed into the body circulation (52). Some absorption enhancers are toxic to Caco-2 cells *in vitro*, but relatively safe in animals probably due to the repair mechanism in intact mucosal tissues. Some absorption enhancers have reversible open tight junction between act by reversible open, short-term use smaller side effects, such as cationic chitosan derivatives. But when treating chronic diseases such as diabetes or osteoporosis, we must pay attention to these toxic effects of long-term oral materials. (54).

### Enzyme Inhibitors

Protein and peptide drugs are easily degraded by various enzymes in the gastrointestinal tract. Therefore, enzyme inhibitors can effectively prevent the enzymatic degradation of protein and peptide drugs and increase drug absorption. Research shows that after oral application of enzyme inhibitors, the oral bioavailability of protein and peptides drugs have been significantly improved (33). Liu H *et al* (55) reported effects of five protease inhibitors (including leupeptin, sodium glycocholate, bacitracin, bestatin, and cystatin) on the intestinal absorption and degradation of insulin in rats, and found that these protease inhibitors could increase the insulin efficacy more effectively in the large intestine than in the small intestine.

The enzyme inhibitors and drugs can be encapsulated in the nanoparticle system at the same time, which can protect the drug from enzyme damage more effectively, and improve the gastrointestinal absorption of polypeptide protein drugs. However, the use of enzyme inhibitors may also cause the polypeptide or protein that should be normally degraded to be absorbed by the intestine. One of the major disadvantages of these inhibitors is that they have high toxicity, especially in the long-term use of the drug (56). And inhibition of enzymes in the gastrointestinal tract by long-term use of enzyme inhibitors may interfere with the normal digestion and absorption of protein and may bring reversible or even irreversible damage to the structure and function of the human gastrointestinal tract and impair digestion of nutritive peptides and proteins (53,57). The bioavailability of protein and peptide drug is enhanced by the combination of enzyme inhibitors and absorption enhancers (58). However, the security of the two combined use remains to be further studied. What’s more, enzyme inhibitors are specific and only play a role at a certain time and at certain sites. And drugs and enzyme inhibitors must simultaneously pass through the metabolic sites. The presence of enzyme inhibitors will affect the normal absorption of gastrointestinal nutrition, and may even generate feedback regulation to stimulate excessive secretion and expression of enzymes. Long-term treatment will lead to splenic hypertrophy and hyperplasia.

### Mucoadhesive

Although the mucous layer may interfere with drug absorption, the presence of the mucus layer is beneficial to the preparation of the bioadhesive mucosal drug delivery system (59). The biological adhesive drug delivery system mainly uses mucous membrane between the polymer material and mucus to produce mucous membrane adhesion, increase drug residence time in mucous membrane, and improve the efficacy of drugs (33). And, mucosal adhesion nanoparticles extend the retention time of gastrointestinal tract by electrostatic interaction, hydrophobic interaction, van Edward force and polymer chain interaction and penetration, thereby enhancing drug absorption (60). Mucous adhesives can directly change the permeability of mucous epithelium and improve the bioavailability of protein and peptide drugs (61). The mucosal adhesion system can also inhibit the degradation of protein peptides, enhance the stability of protein peptides, open the tight junctions between epithelial cells, increase the transmembrane permeability of protein peptides, control the release rate of protein polypeptides, and reduce the frequency of administration (59). It was reported that mucous adherence nanoparticles (MNP) may be a suitable nano-carrier for protein and peptide drugs, due to the increased retention time of MNP in the GI tract to promote absorption and it easily attaches to the mucus layer to increase the concentration gradient of the drug (62–64). Jianyong Sheng *et al* (62) prepared insulin low molecular weight protamine MNPs. After oral administration, it showed a sustained hypoglycemic effect with a faster onset in diabetic rats, and the pharmacological availability of orally delivered conjugate-loaded MNPs was 17.98 ± 5.61% relative to subcutaneously injected insulin solution, with a 2-fold higher improvement over that by MNPs loaded with native insulin after oral administration.

Some studies have shown that chitosan has a positive charge, and the mucous layer is negative, that is, chitosan has mucous adsorption, which can prolong the retention time of the drug in the small intestine (65). In addition, chitosan opens the tight junction of Caco 2 monolayer cells by invertibility, reducing its transmembrane resistance and enhancing the permeability of the cell bypass (66). Mukhopadhyay *et al* (67) prepared polyamide -chitosan nanoparticles with the sensitivity of pH protecting drug invariance in the gastrointestinal tract; In oral administration of diabetic mice (50 IU/kg), the relative bioavailability was 11.78%, and no serious systemic toxicity. pH-sensitive nanoparticles, poly methacrylic acid chitosan polyethylene glycol, can be used for the oral delivery of insulin. The encapsulation efficiency of the nanoparticles can reach 99.9%, the average particle size is 172 nm, and the release of insulin in the intestine is two times that in the stomach (68). Su *et al* (69) prepared two ethylenediamine, five acetic acid, and polyglutamic acid chitosan nanoparticles. When pH value was > 7, nanoparticles gradually expanded and degraded. By opening the tight junctions of intestinal epithelial cells, the absorption of insulin in the small intestine could be promoted, and the time of hypoglycemia could be prolonged.

In recent years, more and more mucosal adhesion systems have been applied to the oral administration of insulin, salmon calcitonin, Essen, octreotide, thymic five peptides, vaccine, and cyclosporin A. The mucosal adhesion and drug delivery system significantly improved the pharmacokinetics and efficacy of these proteins and peptides. However, the mucosal drug delivery system still has some limitations, and this is because the adhesion between the mucous adhesion system and the mucous layer is achieved through the interaction with the mucin fiber (70). The update time of mucin in intestinal mucus is 50~270 min. Therefore, a mucoadhesive polymer residence time in the intestine is only 4~5 h; and the mucoadhesive system and the mucus layer adhered tightly together, so that the mucoadhesive system cannot through the mucus layer into epithelial cells (71). The present study focused on the mucoadhesive nanoparticles to protein and peptide drugs safely and effectively to the small intestine, the rapid drug release in small intestinal mucus layer, intestinal epithelial cells and then open the close connection between nanoparticles enter the circulation.

### Colon-Specific Drug Delivery

Protein and peptide drugs may be degraded under the condition of partial acidity or partial alkalinity, so the intestinal release in theory in a near-neutral environment is beneficial to protect the activity of protein and peptide drugs (53). At the end of the gastrointestinal tract in the colon, the first-pass effect is avoided since the enzyme activity in the colon is low and is conducive to the absorption of protein and peptide drugs; and the transit time of solid preparation of nanoparticles in the colon can reach 20~30 h, which is a controlled-release research potential formulation.

The colon-targeting nanoparticle delivery system mainly relies on a pH-dependent vector. With the change of pH value in the gastrointestinal tract, the release of protein drugs in the stomach and small intestine is reduced, and the protective protein reaches the colon area to achieve the best effect. However, the dosage form also has some limitations, because in many cases, especially for patients with gastrointestinal lesions, gastrointestinal pH values are different from those of normal people. At the same time, the time of the drug’s arrival in the colon is affected by food. The change of food species and the size of food will change the transit time in the gastrointestinal tract, so the individual difference is obvious, and it cannot be individualized, which leads to the decrease of the bioavailability of the preparations.

There are some specific areas of the small intestine that are immunity related, such as Peyer’s patches to lymphokines and some particles into the circulatory system; the use of enteric-coated controlled-release technology or other drug releases in the small intestine may increase the absorption of protein and peptide drugs.

## OTHER METHODS TO PROMOTE THE ORAL DELIVERY OF PROTEIN AND PEPTIDE DRUG NPs

### Modification of Target Molecules or Perssad

The target nanoparticles are prepared on the surface of the nanoparticles on the surface of the target molecule or target group. By targeting the recognition of molecules or groups, nanoparticles can specifically bind to the corresponding receptors on the surface of small intestinal epithelial cells, thereby reducing the entrapping and scouring effects of mucus on nanoparticles and increasing the absorption of drugs. The commonly used target molecules include lectin, invasive element, and vitamin B12. Yin (72) thinks that lectin can interact with the mucin glycoprotein complex, triggering the phagocytosis of intestinal epithelial cells, and achieving the uptake of nanoparticles in intestinal epithelial cells.

### Ligand Modification

Ligand modification strategy has become one of the hotspots of oral protein peptide delivery system in recent years. Lectin is covalently bound to nanoparticles *via* interaction with mucus or the intestinal epithelial cell surface, which assists nanoparticles pass through the intestinal mucosa (5,48). Another hot protein ligand is RGD (a short peptide containing arginine glycine aspartic acid), which can bind to the specificity of the β1 binding protein on M cells (49). After the RGD peptide was added to the nanoparticles, the nanoparticles could penetrate the M cells to a large extent, thus covalently binding polyethylene glycol onto the RGD, which contributed to the targeting of nanoparticles (50).

Integrin is a group of transmembrane glycoproteins on cell membranes. It is a heterodimer composed of alpha and beta subunits through non-covalent bonding (73). It can be expressed on a large number of cell surfaces, such as vascular endothelial cells, epithelial cells, and myocytes, and there are many integrins on the surface of some cells (74). Many integrin receptors represented by alpha v beta 3 have a common feature: they can interact with extracellular matrix proteins through three amino acid sequences of arginine, glycine, and aspartic acid (RGD) (75). And peptides containing RGD sequences have integrin receptor targeting. Anti-tumor strategies based on RGD have been extensively studied in the field of diagnosis and treatment of cancer. However, there are few reports on the application of RGD in the field of oral administration of protein and polypeptide drugs. Therefore, the authors envisage whether RGD peptide can be linked to the carrier material, and the interaction between RGD and integrin receptor can make the delivery system targeted to intestinal epithelial cells, so as to improve the oral absorption of protein and polypeptide drugs. cRGDyk is a synthetic cyclic pentapeptide containing RGD sequence. It has a high affinity for integrin receptors and is not easy to degrade. It is a preferred choice for targeted modification of carrier materials (76). Based on the above considerations, a nano-oral delivery system targeting integrin receptors was constructed by using trimethyl chitosan modified by cRGDyk to improve the bioavailability of oral protein and polypeptide drugs.

### Protein Nanocrystallization Technology

In order to extend the shelf-life of protein drugs, protein drugs are usually freeze-dried into solid form. However, freeze-dried protein drugs often exhibit protein aggregation and secondary structure changes, which eventually lead to drug loss of biological activity. Nanocrystallization is one of the ways to maintain high stability and biological activity of proteins. Protein nanocrystallization is a new kind of oral drug delivery system. Protein nanoparticles with a particle size of 50–500 nm are formed by crystallization technology, and stable nanoparticles are formed by charge or space stabilization of surfactants. Protein nanocrystallization can effectively protect the biological activity of proteins, with good biocompatibility, and it is easy to degrade and assemble.

### Nanoparticles Containing Cell-Penetrating Peptides

Cell-penetrating peptides are short peptides composed of positively charged amino acid fragments. They have excellent membrane-penetrating ability and can carry macromolecular substances or nanoparticles into cells. However, the mechanism of cell-penetrating peptides promoting macromolecule uptake is still unclear. Simple physical mixing of some penetrating peptides and insulin can promote insulin absorption in the intestinal mucosa, while other penetrating peptides must covalently bind with insulin in order to play a role in promoting insulin absorption (77,78). Negatively charged insulin and positively charged penetratin form a complex by electrostatic adsorption, and pHPMA is wrapped on the surface of the complex to form nanoparticles (79). The encapsulation of pHPMA makes the surface of the nanoparticles hydrophilic and can cover up the positive charge of penetration, which makes the nanoparticles have a higher efficiency of penetrating the mucus layer. In the process of penetrating the mucus layer, pHPMA gradually separates from the surface of the nanoparticles, releasing the penetratin-insulin complex. The penetration of insulin into intestinal epithelial cells significantly increased the ability of insulin to penetrate intestinal epithelial cells. The absorption of the nanoparticles in epithelial cells secreted by mucus is more than 20 times that of free insulin. The pharmacological availability of the nanoparticles in diabetic rats after intragastric administration is 6.61% relative to subcutaneous insulin injection.

## CONCLUSION AND THE PROSPECT OF PROTEIN AND PEPTIDE DRUGS

The chemical modification of protein polypeptide can improve the stability of protein polypeptide drugs, increase the permeability of the membrane, reduce the immunogenicity, and reduce its bioactivity, and the absorption enhancer can promote the absorption of the small intestine to the protein polypeptide. But it has no protein specificity, while promoting the absorption of drugs; it may also promote the gastrointestinal toxicity. The mucosal adhesion system can prolong the retention time of protein and polypeptide drugs in the gastrointestinal tract and improve its bioavailability, but it cannot increase the oral permeability of drugs and avoid the cleaning of the small intestinal mucosa.

Nanoparticles, which have unparalleled advantages over other methods, are the most studied carriers of oral administration. However, due to the complexity of the nanoparticles, the preparation cost is high and it is not easy to enlarge. At the same time, nano-microspheres are prepared as oral delivery carriers by nanoparticles, preparation methods, particle size, surface charge, hydrophobicity, and loading drugs. Due to many factors such as loading capacity, loading way, and physicochemical properties of drugs, the best combination of factors has not been optimized yet.

Oral administration of safety, as the ideal mode of administration, has become a hot spot of protein polypeptide drug delivery system. Because the types of protein polypeptide are varied, there is no one popular strategy suitable for oral administration. Most of the research on the oral administration system of protein peptides is limited to improving the utilization of oral peptides and peptides and proteins in the body. However, the study on how to control the release rate of the lesions is less. The development of knowledge and oral gastrointestinal disorder of protein absorption of protein and peptide and the mechanism of drug delivery systems and nanotechnology fully integrate and utilize the existing knowledge on the nanoparticles based on a systematic, personalized design from the effects of drug release and absorption of various large computers. It is hopeful to design an oral administration system of proteins and polypeptides that can be used in the clinical practice.

The structure of nanoparticles is relatively stable, which can protect protein drugs from digestive enzymes in the gastrointestinal tract to a certain extent. At the same time, nanoparticles have the characteristics of slow control, which can slow drug release and prolong the time of action. Although oral administration has great advantages over subcutaneous injection, there are still some problems. First, oral protein nanoparticles have low bioavailability relative to subcutaneous injection. Secondly, the oral protein drug nanoparticles were only tested in mice and rabbits, and the data obtained was not applicable to the human body. Taking high-dose protein nanoparticles can achieve long-term effects, but it will promote mitosis of gastrointestinal epithelial cells and have adverse effects on the physiological environment of the gastrointestinal tract.

Finally, with the understanding of the absorption barrier and mechanism of the gastrointestinal tract and the mechanism of protein polypeptide absorption and the development of the oral administration system and nanotechnology of the protein polypeptide, the knowledge is fully integrated and utilized. Based on the nanoparticles, the systematic and personalized design of the drug release and absorption in various aspects of drug release and absorption by large computers is expected. A protein peptide oral delivery system is designed for clinical use.
